# Thalidomide with blockade of co-stimulatory molecules prolongs the survival of alloantigen-primed mice with cardiac allografts

**DOI:** 10.1186/s12865-020-00352-1

**Published:** 2020-04-16

**Authors:** Maoshu Zhu, Yunhan Ma, Kai Tan, Liyi Zhang, Zhaowei Wang, Yongsheng Li, Yingyu Chen, Junjun Guo, Guoliang Yan, Zhongquan Qi

**Affiliations:** 1 Xiang’an Branch, The First Affiliated Hospital of Xiamen University, Xiamen, 361100 Fujian China; 2 The Fifth Hospital of Xiamen, Xiamen, 361100 Fujian China; 3 Organ Transplantation institute, School of Medicine, Xiamen University, Xiamen, 361100 Fujian China; 4 Fujian Key Laboratory of Organ and Tissue Regeneration, Xiamen, 361100 Fujian China; 5 Grade 2015 Clinical Medicine, Fuzhou Medical College of Nanchang University, Fuzhou, 344000 Jiangxi China; 6 School of Medicine, Guangxi University, Nanning, 530004 Guangxi China

**Keywords:** Thalidomide, Co-stimulatory molecule, Monoclonal antibody, Cardiac allograft, Alloantigen, Memory T cell

## Abstract

**Background:**

Miscellaneous memory cell populations that exist before organ transplantation are crucial barriers to transplantation. In the present study, we used a skin-primed heart transplantation model in mouse to evaluate the abilities of Thalidomide (TD), alone or in combination with co-stimulatory blockade, using monoclonal antibodies (mAbs) against memory T cells and alloantibodies to prolong the second cardiac survival.

**Results:**

In the skin-primed heart transplantation model, TD combined with mAbs significantly prolonged the second cardiac survival, accompanied by inhibition of memory CD8^+^ T cells. This combined treatment enhanced the CD4^+^Foxp3^+^ regulatory T cells ratio in the spleen, restrained the infiltration of lymphocytes into the allograft, and suppressed the allo-response of spleen T cells in the recipient. The levels of allo-antibodies also decreased in the recipient serum. In addition, we detected low levels of the constitutions of the lytic machinery of cytotoxic cells, which cause allograft damage.

**Conclusions:**

Our study indicated a potential synergistic action of TD in combination with with mAbs to suppress the function of memory T cells and increase the survival of second allografts in alloantigen-primed mice.

## Background

Memory T cells (Tms) are important immune system cells that protect against pathogen invasion. In adults, memory phenotypes are exhibited by 40–50% of T cells circulating in the peripheral blood [1]. Alloreactive Tms can be developed by transplantation recipients if they were exposed to the alloantigen during previous transplantations, blood transfusions, and pregnancies, or as a result of the continuous exposure to viral pathogens and bacteria. These memory cells play a pivotal role in poor allograft outcomes [2–4]. Compared with naïve cells, Tms have lower activation thresholds, pre-committed cytokine profiles, and fewer dependent costimulations [5, 6], which make them significant obstacle to prolonging allograft survival after initial and secondary transplants. CD4^+^ Tms and CD8^+^ Tms are two important components of Tms. Primed CD8^+^ Tms can continuously infiltrate the allograft within 72 h post-transplantation, and CD8^+^ Tms-produced interferon gamma (IFN-γ) can promote allograft rejection [7]. In secondary lymphoid organs, CD4^+^ Tms are transformed and amplified into effector CD4^+^ T cells, which aid the production of donor-specific antibodies by B cells [8, 9]. With the help of CD4^+^ T cells, the activated alloreactive CD8^+^ T cells and donor-reactive alloantibodies, together with CD4^+^ T cells, cause allograft damage. Thus, basic research is required to better understand the complex immune mechanisms in host sensitization to allo-antigens. Subsequently, much needed novel therapeutic approaches to manage sensitized transplant patients should be developed.

In the laboratory, co-stimulatory blockade, such as monoclonal antibodies (mAbs) have been proved to be high efficiency on naïve T cells in primary organ transplantation [10]. In many mouse transplantation model, Anti-CD154 mAbs combined with anti-lymphocyte function-associated antigen 1 (LFA-1) mAbs can induce tolerance of primary allografts by effectively blocking LFA-1/intercellular adhesion molecule 1 signaling [11–16]. However, in primed-sensitized recipients, mAbs and clinical first-line immunosuppressive agents have limited effects on Tms [17–19].

Thalidomide (TD) was primarily prescribed as a sedative or hypnotic. Afterwards, it was used to treat nausea and to alleviate morning sickness in pregnant women; however, it was quickly withdrawn from the European and Canadian markets in 1961 and 1962 because of its teratogenic effects [20]. However, later, it was found that TD had effects in the treatment of multiple myeloma, erythema nodosum leprosum lesions, and various autoimmune diseases [21]. In recent years, the immunosuppressive and anti-inflammatory effects of TD have been proven in organ transplantation [22–25] and in the treatment of other diseases [26, 27]. Administration of minimal or moderate TD doses can exert immunosuppressive effects to prevent acute cardiac allograft rejection; indeed, its synergism with some clinical medications can significantly improve the survival of heart grafts [24, 25].TD has been described as having a ability on the suppression of tumor necrosis factor-alpha (TNF-α) and the modulation of interleukins [28]. TD increases the degradation of TNF-α mRNA to control its protein level in monocytes and macrophages [29, 30]. Recent studies have suggested that members of the TNFR-TNF superfamily might be crucial for the generation of Tms and the maintenance of high levels of antigen-reactive T cells [31]. These results allowed us to hypothesize that TD might ameliorate acute allograft rejection by acting against memory cells.

In the present study, we firstly assessed TD and its synergistic effect combined with co-stimulation blockade using anti-CD154 and anti LFA-1 antibodies in skin-primed heart transplantation. We then studied TD’s in vivo mechanism of action, which provided a therapeutic breakthrough in clinical organ transplant research.

## Results

### Allograft survival is significantly prolonged in pre-sensitized recipients treated with TD + mAbs

**Fig. 1:**
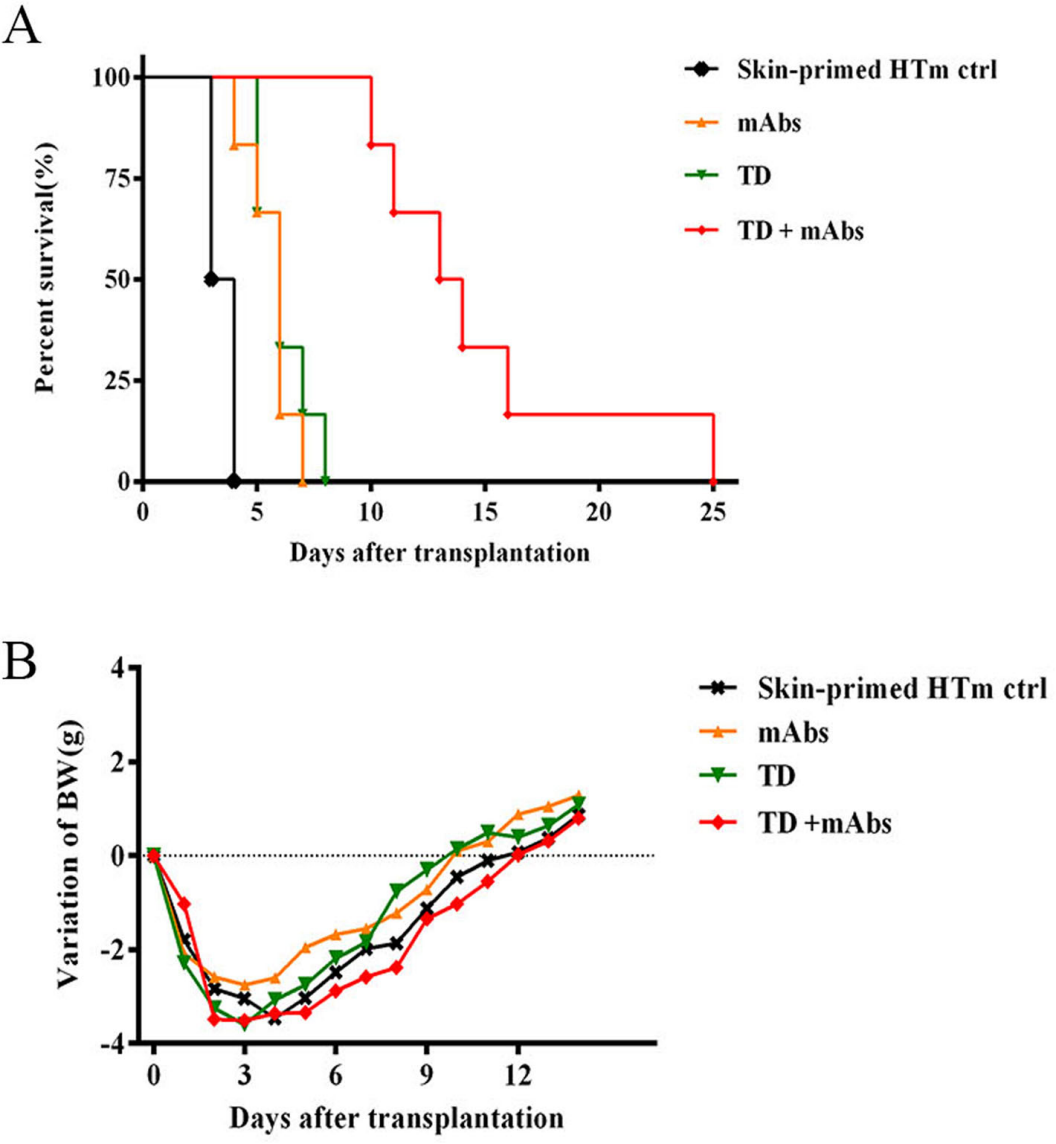
Survival time of cardiac allografts and changes in body weight in mice receiving allografts after transplantation. Heterotopic vascularized hearts from BALB/c mice were transplanted into skin-primed C57BL/6 recipients. **a** Graft survival time shown as a Kaplan–Meier curve for the four represented sets (control, TD group, mAbs, and TD + mAbs groups). The MST of these four groups were 3.5 ± 0.5, 6 ± 1.1, 6 ± 0.9, and 13.5 ± 4.9 days, respectively. **b** Body weight change curve of the recipient mice. Trends of the four different sets were similar indicating almost no differences among them

Our previous research showed that BALB/c hearts would be rejected within 7 days (mean survival time) in naïve wild-type (WT) C57BL/6 recipients; however, rejection was accelerated to within 4 days in mice sensitized using BALB/c skin [32]. Thus, we investigated whether the accelerated rejection response could be diminished using TD alone or combined with mAbs in a skin-primed heart transplant model (HTm). C57BL/6 mice that were pre-sensitized with BALB/c skin were transplanted with hearts from BALB/c donors, and the recipients were randomly allocated into four groups (based on the treatment protocol shown in Table 1), with six mice in each group. As displayed in Fig. 1a, in comparison with the control group (3.5 ± 0.5 days), there was a significant extension in survival time of the cardiac graft after mAbs (6 ± 0.9 days) and TD (6 ± 1.1 days) treatments (*P* < 0.05). The treatment effect was further enhanced by treatment with TD + mAbs, which improved the mean survival time (MST) of the grafts to 13.5 ± 4.9 days (*P* < 0.01). Thus, TD showed synergistic effects when combined with costimulatory blockade in suppressing secondary cardiac allograft rejection.

**Table 1:** Experimental treatment groups

Skin-primed HTm model	Reagent	Treatment
Control	Saline	Anti-LFA-1 (0.1 mg) and anti-CD154 (0.25 mg/d) were administered i.p. on days 0, 2, and 4 after transplantation. TD (100 mg/kg/day) was administered i.g. on days 0–10 after transplantation, the same as the saline.
mAbs	anti-LFA-1 and anti-CD154	
TD	TD	
TD + mAbs	TD, anti-LFA-1, and anti-CD154

Another important consideration is the effect of drug toxicity on the graft recipients. To explore this question, body weight changes after heart transplantation were analyzed. After drug treatment, we found there was a small change in body weight, which could probably be explained as a reaction to the surgical trauma (Fig. 1b). Taken together, these data suggested that the allograft survival time could be significantly extended (*P* < 0.01) without obvious adverse reactions.

### TD + mAbs decreased the proportions of Tms and the functions of lymphocytes, but increased Tregs in skin-primed recipients during the secondary heart transplantation

**Fig. 2:**
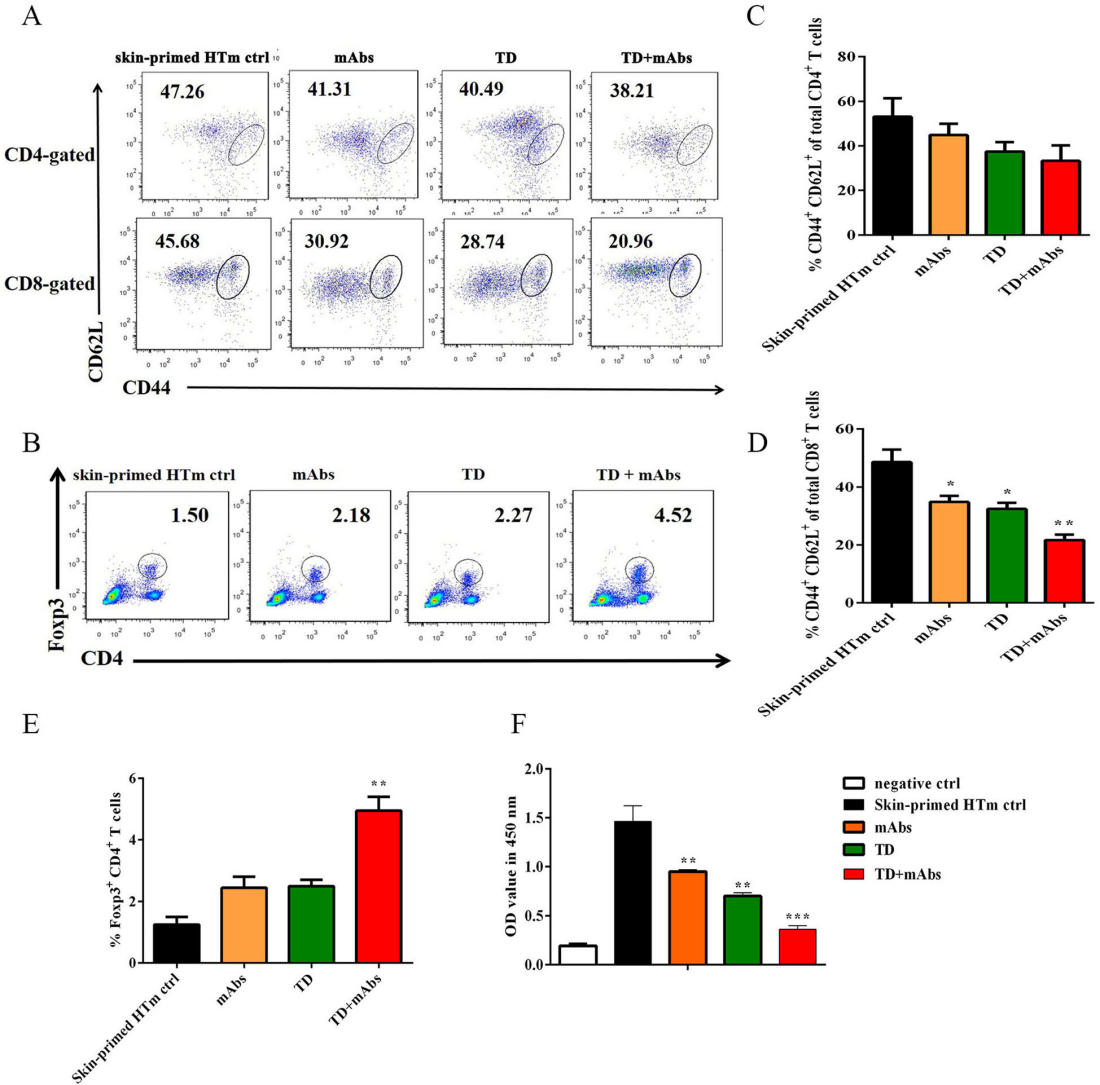
Splenic lymphocyte function and Tms and Tregs proportions in skin-primed recipients on day 4 after the secondary heart transplant. **a** CD4^+^ Tm/CD4^+^ T cell proportions (top), CD8^+^ Tm/CD8^+^ T cell proportions (bottom); **b** Flow cytometry analysis of Tregs; Cumulative data analysis for the proportion of **c** CD4^+^ Tm/CD4^+^, **d** CD8^+^ Tm/CD8+ T cells and **e** Tregs (*n* = 3 mice/group); **f** Proliferation of recipient splenic T cells in response to donor BALB/c cell as assessed using MLR assay. Among the groups, the mean OD values were compared (*n* = 3 mice/group). Splenic T cells alone served as the negative control. Data are representative of three separate experiments (*, *P* < 0.05; **, *P* < 0.01; ***, *P* < 0.001 versus skin-primed HTm ctrl)

For alloantigen-primed mice, Tms exist primarily in the spleen and lymph nodes. Homing of effector Tms, especially splenic Tms, is pivotal in the rejection of secondary transplantation [33, 34]. Therefore, the memory phenotype of the recipient splenocytes 4 days after heart transplantation was investigated. In contrast to the skin-primed HTm control group, the treatment groups did not show markedly different proportions of memory CD4^+^ T cells in the spleens of the recipients (*P* > 0.05, Fig. 2c). In all treatment groups, there was a significant decrease in the proportion of memory CD8^+^ T cells among CD8^+^ T cells, and this decrease was significantly (*P* < 0.01) enhanced the TD + mAbs group (Fig. 2a, d). Meanwhile, the CD4^+^Foxp3^+^ Tregs proportion among recipient splenocytes increased in all treatment groups, with a significant increase in the combined treatment group (*P* < 0.001) (Fig. 2b, e).

The spleens of the recipient mice were harvested on day 4 after transplantation, and to perform mixed lymphocyte reaction (MLR) assays, splenocytes were prepared to assay the alloreactivity of lymphocytes. As shown Fig. 2f, in contrast to the skin-primed HTm control group, the groups receiving TD or mAbs monotherapy (*P* < 0.01) both showed effective suppression of splenocyte alloresponses. Notably, TD showed a marked synergistic effect in combination with mAbs to restrain the alloresponses of the splenocytes compared with that of the skin-primed HTm control group (*P* < 0.001).

### The combined treatment group showed an intact myocardial structure and fewer inflammatory cells infiltrating the graft

**Fig. 3:**
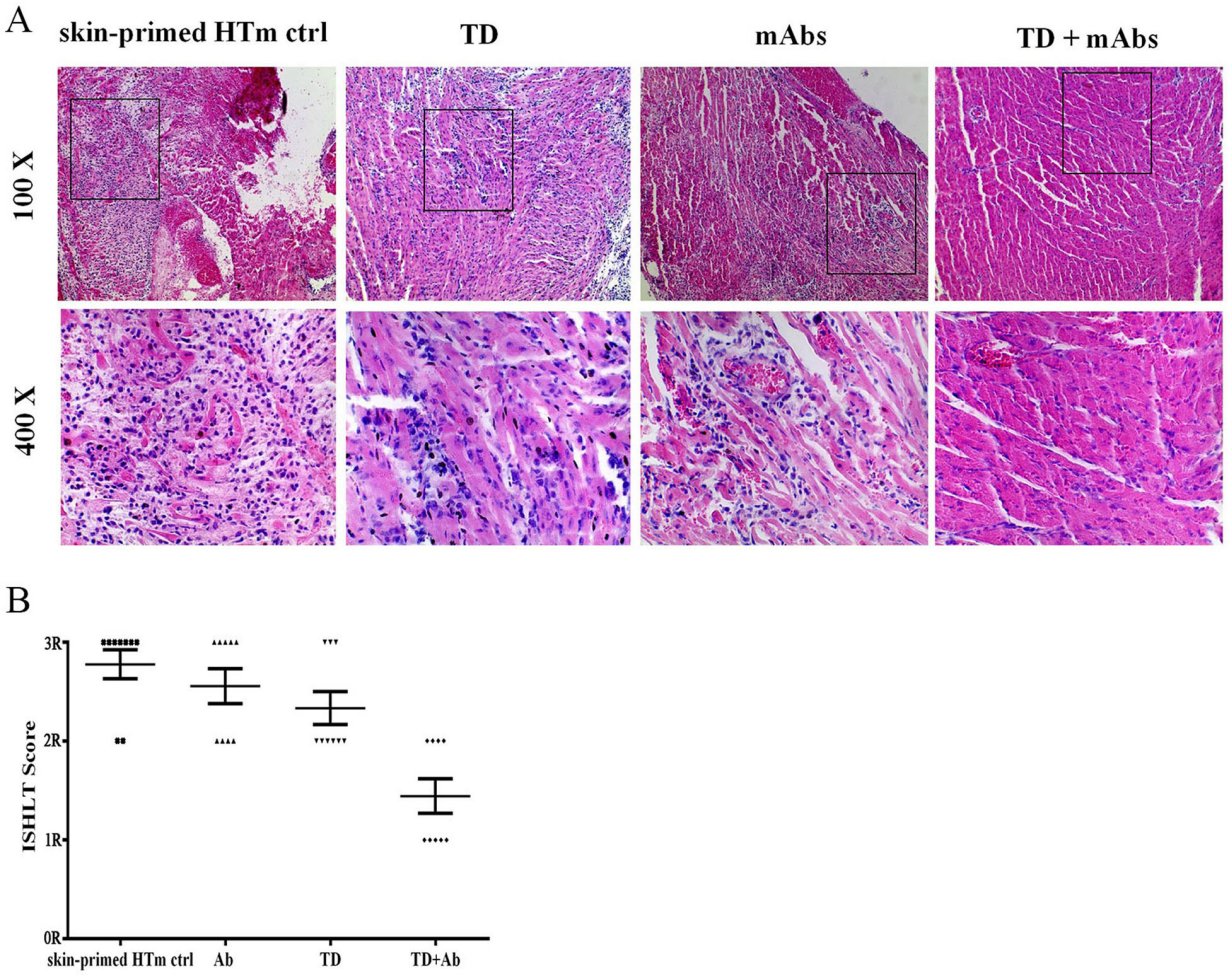
Histological evaluation**.** On day 4 post transplantation, the grafted hearts were harvested. Histological evaluation was performed on the same parts of each graft. **a** The microscopic images heart tissues stained with H&E are shown at 100× (top) times and 400× (bottom) magnification under a light microscope. **b** The data show the ISHLT scores for the hearts. The scores for each animal in each group are represented by dot plots. The mean scores are represented by the line (*n* = 3; *, *P* < 0.05; **, *P* < 0.01; ***, *P* < 0.001 versus skin-primed HTm ctrl)

Next, we investigated the influence of the combined treatment on day 4 after heart transplantation. Allografts from each group were subjected to histological sectioning and hematoxylin and eosin (H&E) staining. As shown in Fig. 3a, in the control group, the allografts showed extensive necrosis of myocardial cells, massive infiltration of inflammatory cells, and a large amount of thrombus. Contrastingly, the grafts from the mAbs and TD groups showed moderate inflammatory cell infiltration and tissue damage, and a smaller amount of thrombus. In contrast to the above allografts, in the grafts from TD + mAbs group, the myocardium was very well preserved, with no evidence of degeneration, destruction, or inflammatory cells infiltration. The overall scores of rejection/inflammation were measured using the International Society for Heart and Lung Transplantation (ISHLT) ranking (Fig. 3b). The rankings given for the TD + mAbs group were significantly lower than those for the skin-primed HTm control group (*P* < 0.05 in each comparison). This result indicated that TD combined with blockade of co-stimulatory molecules provided enhanced protection from rejection Treatment with TD could provide some protection against accelerated rejection; however, when combined with co-stimulatory molecules blockade, this limited protection was promoted significantly.

### Production of rejection and tolerance-related cytokines in the allografts and recipient sera

**Fig. 4:**
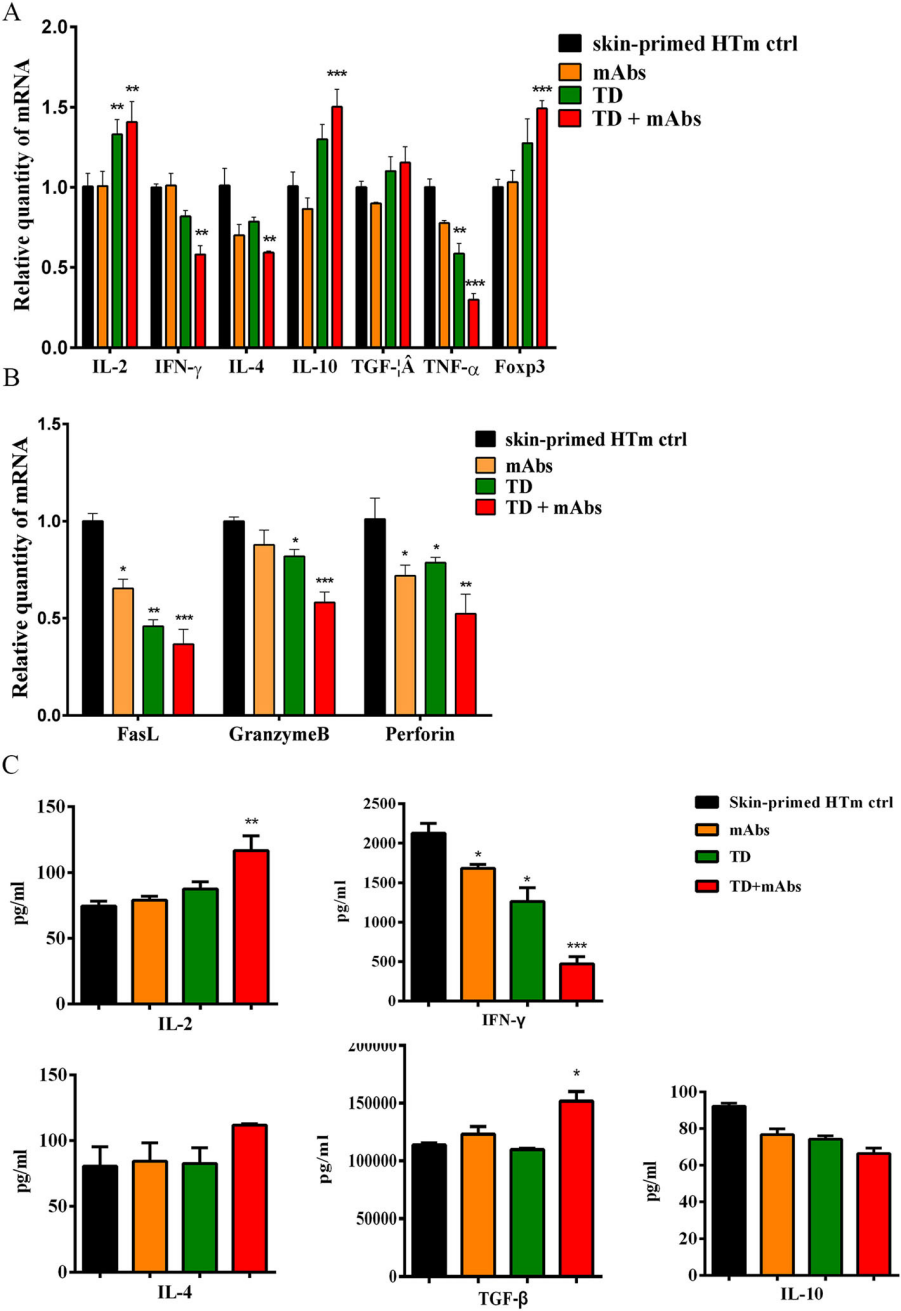
The expression levels of rejection and tolerance-related cytokine genes in allografts were detected using qRT-PCR and the levels of rejection and tolerance-related cytokines in the sera of the recipients were detected using ELISA. On day 4 post-transplantation, allografts and sera were harvested from the skin-primed recipients. **a** Expression levels of the genes encoding IL-2, IFN-γ, IL-4, IL-10, TGF, TNF-α, and Foxp3 within the allografts. **b** Expression levels of the genes encoding Fasl, Granzyme B, and Perforin within the allografts. **c** The protein levels of IL-4, IL-2, IFN-γ, TGF-β, and IL-10 in the recipient sera. The reactions were repeated three times and the data represent three separate experiments (*n* = 3 mice per group; *, *P* < 0.05; **, *P* < 0.01; ***, *P* < 0.001 versus skin-primed HTm ctrl)

To investigate the mechanism by which TD provides graft protection, total RNA was extracted from the allograft and quantitative real-time reverse transcription PCR (qRT-PCR) was used to determine the relative expression levels of cytokines. Figure 4a shows reductions in the relative mRNA expression levels of the genes encoding tumor necrosis factor alpha (TNF-α), interferon gamma (IFN-γ), and interleukin (IL)-4 in all treatment groups. The expression levels of these genes were dramatically lower in the TD + mAbs group compared with those in the skin-primed HTm control group (*P* < 0.001, *P* < 0.01, and *P* < 0.01, respectively). The relative expression levels of IL-2 mRNA were significantly enhanced in the TD and TD + mAbs groups (*P* < 0.01). IL-10 and forkhead box P3 (FOXP3) mRNA relative expression levels were significantly increased in TD + mAbs group (*P* < 0.001).

There is a consensus in clinical research that heightened expression levels of the genes encoding the cytotoxic molecules perforin and granzyme B within the allograft are characteristic of acute rejection. As shown in Fig. 4b, the relative expression levels of perforin and granzyme B were significantly reduced in the TD + mAbs group compared with skin-primed HTm control group (*P* < 0.01; *P* < 0.001, respectively). Compared with the TD, mAbs monotherapy group, TD combined with mAbs show a synergistic effect on the relative expression levels of perforin and granzyme B mRNA. We also assessed the levels of the mRNA encoding the effector molecule FasL, which induces target cell death via cytotoxic T lymphocytes. Compared with skin-primed HTm control group, the expression of FasL was significantly decreased in all treatment groups.

At 4 days after heart transplantation, the levels of rejection and tolerance-related cytokines (IL-2, IL- 4, IFN-γ, IL-10, and TGF-β) in the recipients’ serum were analyzed using an enzyme-linked immunosorbent assay (ELISA) (Fig. 4c). Compared with their levels in the sera of the skin-primed HTm control group, the combined treatment enhanced the level of IL-2 and TGF-β, and reduced the level of IFN-γ significantly (*P* < 0.01; *P* < 0.05; *P* < 0.001, respectively), However, the serum levels of cytokines IL- 4 and IL-10 remained unchanged among the four groups.

### TD + mAbs decreased humoral immunity in the skin-primed HTm

**Fig. 5:**
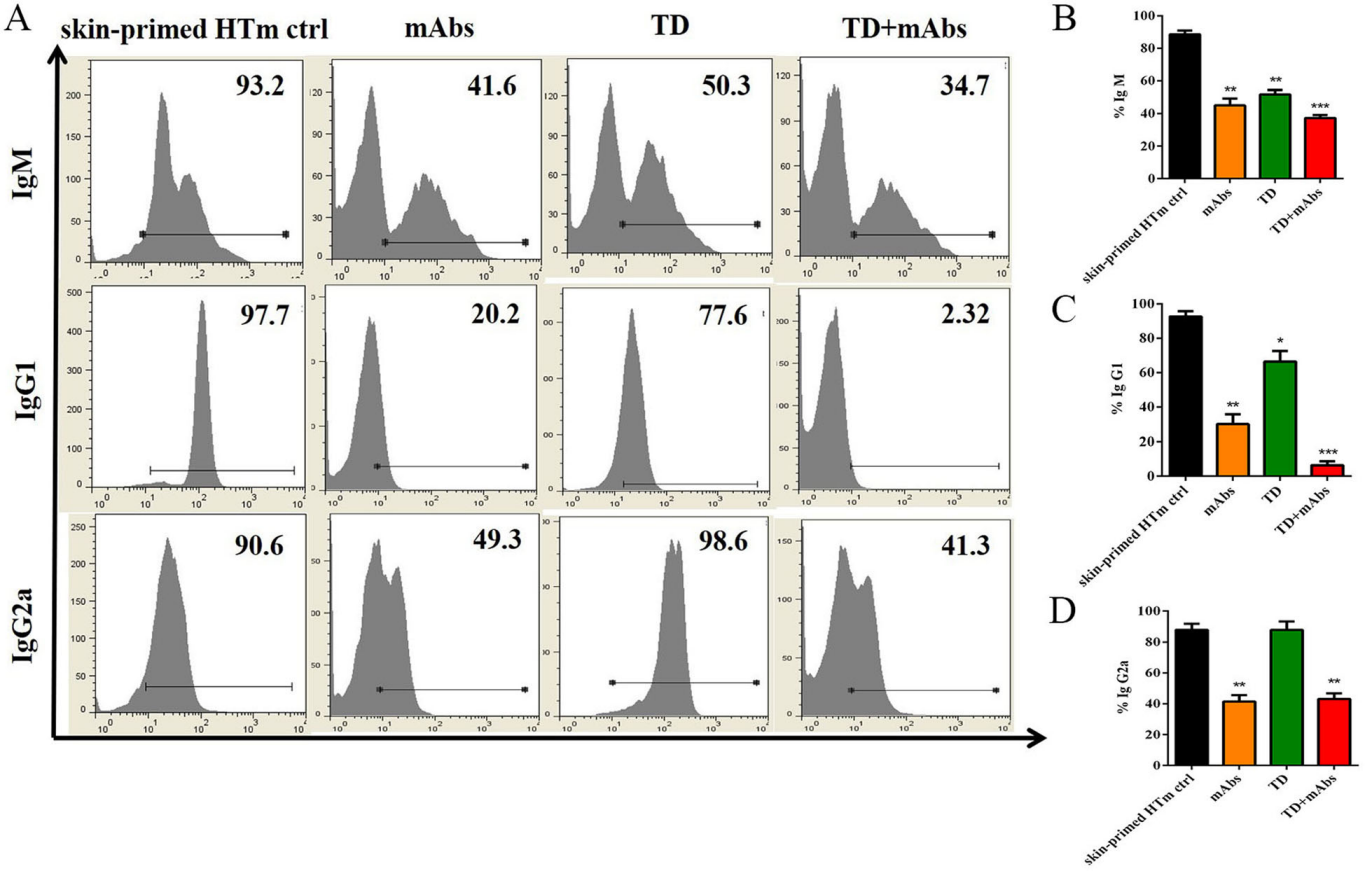
Serum levels of alloantibodies in skin-primed recipients on day 4 after the secondary heart transplant. **a** Flow cytometry analysis of Ig M (top), Ig G1 (middle), Ig G2a (bottom); **b** Cumulative data analysis for the proportion of **b** Ig M, **c** Ig G1 and **d** Ig G2a (*n* = 3; *, *P* < 0.05; **, *P* < 0.01; ***P < 0.001 versus skin-primed HTm ctrl)

Flow cytometry was used to detect the production of donor-specific antibodies at 4 days after heart transplant in the skin-primed recipients. At this time point, the control recipient mice showed high IgG and IgM levels. As shown in Fig. 5, mAbs has limited effects on the production of donor-specific antibodies, and in combination with TD, they could further enhance the inhibition of antibody production (Fig. 5b, c). TD alone had no effect on IgG2a production; however, when combined mAbs, an obvious reduction in IgG2a levels was observed compared with skin-primed HTm control group.

## Discussion

In the present study, we showed the immunosuppressive effect of TD alone or combined with clinical costimulatory blockade in a clinically relevant pre-sensitized mice cardiac transplantation model. The dosage of TD used was 100 mg/kg/day, whereas, previous studies generally used TD at 0–10 mg/kg/day without toxic effects [24, 25]. However, we observed that TD at 200 mg/kg/day had no toxic effects but further extended the survival time of cardiac allografts in skin-primed HTm, and did not affect the recipients’ body weight (*n* = 12 for each group, data not shown). Therefore, TD at 100 mg/kg/day TD was chosen for the present study. The results showed that treatment with TD combined with mAbs had synergistic immunosuppressive effects to prolong allograft survival compared with that observed in non-or mono-therapy recipients.

The emergence of memory cells before transplantation causes damage to allografts by mediating transplant rejection and blocking the induction of transplant tolerance [8, 25, 35]. In our study, treatment had no effect on memory CD4^+^ T cell, although TD + mAbs could significantly decrease the level of IFN-γ. This phenomenon confirmed that IFN-γ makes a critical difference in maximizing the function of T cells [36]. Thus, TD may weaken the Tms that are derived from effector T cells, which could be explained by the MLR. This finding contributes to our understanding of the mechanism of graft rejection in mice that lack or block CD154, because memory CD4^+^ T cells can mediate allograft rejection [37]. Compared with their naïve counterparts, Tms may have differential requirements for co-stimulation signal pathway blockade, allowing them to easily escape the co-stimulation blockade, and thus contribute to acute or subacute rejection [17]. In our study, compared with monotherapy treatment, TD combined with mAbs effectively decreased the proportion of CD8^+^/CD44(high)/CD62L^+^ T cells in recipient spleen. This might be the dominant effect of the combined treatment in resisting rejection. The synergistic effect of monoclonal antibodies and TD might reflect a suppression of memory T cells by TD and an inhibition of naïve T cell reaction to the alloantigen by monoclonal antibodies. Here, combined treatment could prolong allograft survival owing to these two treatments affected different T cell groups in the re-transplantation mouse model. The in-depth mechanism for the interaction of the two treatment or the crossover point of the two pathways should be further identified.

Several mechanisms of combined treatment might involve in protecting allografts in skin-primed HTm models. The Fas-FasL (Fas ligand) system is significantly associated with programmed cell death [38]. FasL is an effector molecule that is involved in cytotoxic T lymphocyte killing of target cells [39, 40]. Therefore, the Fas-FasL axis seems to act as an effector for cytotoxic T lymphocyte-mediated killing of virus-infected or cancer cells, similar to the perforin-granzyme axis [41]. In our study, combined treatment significantly reduces the expression of FasL mRNA, which is the same as perforin and granzyme B, these might be another mechanism for prolonging graft survival, but should be further verification.

Compared with other groups, the heart allograft tissues from skin-primed recipients receiving the combined treatment showed lower levels of inflammatory infiltration and less damage to myocardial structure. The results of qRT-PCR showed that the combined treatment could significantly reduce the levels of the rejection-related protein IFN-γ within the allograft and in the recipient serum, which implied that the combined treatment inhibited the IFN-γ-related effector function of infiltration T cells. TD combined with mAbs also synergistically upregulated the expression of FOXP3 in the allograft. These two effects protected the allograft and promoted long-term survival.

Flow cytometry showed that the fraction of CD4^+^FOXP3^+^ regulatory T cells among splenic T cell increased after the combined treatment. These results indicated that TD combined co-stimulation blockade protected the allograft by promoting the amount of Tregs. The expression of TGF-β was upregulated in the serum after combined treatment. We hypothesized that increased population of Tregs induced by TD might be TGF-β producing Th3 cells (T helper type 3 cells), which overlap with naturally occurring Tregs, have been identified as regulators in oral tolerance [42]. The tolerance induced by IL-10-secreting T regulatory cells 1 (Tr1 cells), which have been proven to suppress antigen-specific immune responses and to downregulate the pathological immune response in vivo [43]. However, the expression levels of IL-10 in recipient sera did not differ among the treatment groups. Above all, TGF-β promotes the production of Tregs, which would help to prolong allograft survival.

Several studies have reported that costimulatory blockade alone induces allograft immune tolerance by mediating regulatory T cell production, but has no effect on Tms-mediated immune rejection [44, 45]. Tms produce effector cytokines in situ to replenish extra immune cells that can mediate early graft tissue damage. IFN-γ is generally considered a characteristic proinflammatory molecule that is associated with destructive allograft immunity. In the present study, the ELISA and qRT-PCR results indicated that TD, mAbs, and TD + mAbs treatments could significantly reduce the secretion of IFN-γ. Specifically, IFN-γ limits CD4^+^ regulatory T cell expansion and decreases CD25 and FOX3 expression [36, 46]. Tregs are very sensitive to IL-2, and are expanded when conventional T cells produce IL-2 [47], which suggested that combined treatment might induce the robust production of IL-2 by cytotoxic CD4 Th1 cells, thereby enabling expanded Tregs to express more CD25 and FOXP3.

Memory B cells are likely to be a critical cause of eventual rejection, even when Tms are suppressed. Memory B cells have a preferential growth advantage over naïve B cells when activated and during proliferation, as well as being converted to alloantibody secreting plasma cells during the secondary response, making it possible to prevent the adoption of anti-CD154-mediated grafts [2, 48]. In our study, the mAbs and combined treatment decreased IgM/IgG1/IgG2a alloantibodies production in the recipient mice, which was consistent with the results of a previous study showing that treatment with anti-CD154 could decrease the production of alloantibodies [49]. The results suggested that this suppression contributed to prolonging the survival of secondary cardiac allografts.

In our study, combined treatment regimen did not significantly extend the survival period. There are several explanations for this phenomenon. First, the combined treatment could not dramatically affect the proportion of memory CD4^+^ T cells. When the drugs were withdrawn, the Tms cells were activated, proliferated, and converted to effector T cells, and the subsequent restoration of IFN-γ levels would maximize the function of T cells, which could facilitate B cell secretion of allo-antibodies. Secondly, memory B cells are deemed to have priority in interacting with Tms, which can secrete the three cytokines IL-2, IL-4, and IL-10 simultaneously [50]. In the presence of low CD40 levels, compared with naïve B cells, memory B cells show an enhanced ability to differentiate into immunoglobulin secreting cells [48]. Memory B cells can be influenced by the low concentration or by withdrawal of the immunosuppressant, and in the secondary response, cells will be active, proliferative, and converted into alloantibody secreting plasma cells. Thus, continuous administration the regimen could be used as a supplement in a follow-up experiment.

## Conclusions

In summary, the results of the present study demonstrated the potential of TD in the field of organ re-transplantation, suggesting that TD could be used as an effective supplement in clinical immunosuppressive application. Impaired memory CD8+ T cells reproduction or effector T cell function, upregulated levels of TGF-β-producing Tregs, inhibition of cytotoxic effector cell function, and inhibition of inflammatory cell infiltration into the allograft could be included in the machinery of allograft protection. Clinical experiments to reveal the mechanisms by which TD extends the survival of secondary cardiac allografts are currently in progress. Future studies will also seek to identify the signal pathways that regulate the expression and function of the Fas-FasL and perforin- granzyme B systems in the cytotoxic effector cells.

## Methods

### Animals and drugs

Female BALB/c (H-2d) and C57BL/6 (B6, H-2b) mice, aged 8–12 weeks old, were bought from the Slac Laboratory Animal Co. Ltd. (Shanghai, China). C57BL/6 were used as graft recipients and BALB/c (H-2d) as donors. A specific pathogen free facility was used to breed and maintain the mice. The mice were sacrifice by inhaling CO_2_ after experimentation. The experiments were performed in accordance with the guidelines of the Animal Care and Use Committee and Ethics Committee of Xiamen University (Committee’s reference number: XMULAC20170243).

TD (CAS Number: 50–35-1) was purchased from Sigma-Aldrich Chemical Co. USA. Antibodies produced by Bioexpress (West Lebanon) were administered to the mice: anti-CD154 (MR-1), anti-LFA-1 (M17 / 4), and their isotype controls.

### Skin-primed murine heart transplantation model (skin-primed HTm)

Full-thickness, circular trunk skin tissues with a diameter of 1.2 cm from BALB/c mice were engrafted onto the lumbar region of C57B6 mice. Alloantigen-primed mice were defined as those recipients that rejected the BALB/c skin at 4 weeks post-transplantation. Vascularized heterotopic hearts were transplanted into C57B6 recipients from Balb/c donors, using anastomosis to the vessels of the neck with a previously described non-suture cuff technique [51, 52] at 4 weeks post-skin grafting. Graft survival after transplantation was monitored using twice-daily palpation. For 15 consecutive days, body weights were measured daily. The complete loss of a palpable heartbeat in the neck was defined as rejection.

### Treatment protocol

Treatments for the various drugs are summarized in Table 1.

### Histological analysis

Transplanted hearts were harvested on day 4 after transplantation, fixed in 10% neutral buffered formalin, embedded in paraffin, bisected lengthwise, and cut into 5-μm sections for H&E staining using routine methods. Histological evaluation was done using a score modified from the ISHLT [53]. The grades were defined as follows: 0R = no rejection; 1R (mild rejection) = evidence of perivascular infiltrate, interstitial infiltrate, or both with up to 1 focus of myocyte damage; 2R (moderate rejection) = two or more infiltrate foci with related myocyte damage; 3R (severe rejection) = the infiltrate was diffuse and had multifocal myocyte damage ± edema, ± hemorrhage, ± vasculitis. Two pathologists who were blinded to the treatment modalities performed the pathological evaluations.

### Mixed lymphocyte reactions (MLR)

A standard one-way MLR was performed, as described previously [32]. Briefly, nylon wool columns (Wako, Osaka, Japan) were used to isolate splenic cell suspensions from the spleens of C57B6 mice for use as responder cells. Spleen cells from the donors were used as stimulator cells, which were treated with 40 lg/mL mitomycin (Amresco, Solon, OH, USA) before being subjected to the MLR assay. Proliferation assays used stimulator cells (10^5^) cultured with responder cells (5 × 10^5^) in Roswell Park Memorial Institute (RPMI) 1640 medium with 10% fetal bovine serum, 1% penicillin, and 1% streptomycin in 96-well plates. Negative controls comprised responder cells grown in medium lacking the stimulator cells. The cells were mixed and incubated at 37 °C for 72 h in a 95% humidified air with 5% carbon dioxide. After 72 h, cell proliferation was measured using the 5′-bromodeoxyuridine (BrdU) method (Roche, Germany). The percent inhibition values were calculated with respect to the negative control and stimulated control values. The measurements were performed in triplicate.

### Flow cytometry

Lymphocytes were isolated from the spleens of the recipient mice on day 4 after transplantation for flow cytometry analysis. Lymphocytes were labeled using the following antibodies, all of which were obtained from Biolegend (San Diego, CA, USA): Fluorescein isothiocyanate (FITC)-conjugated anti-CD4 (GK1.5), FITC-conjugated anti-CD8 (53–6.7), Phycoerythrin (PE)-conjugated anti-CD44 (IM7), PECy5-conjugated anti-CD62L (MEL-14), PE-conjugated anti-IgM (RMM-1), FITC-conjugated anti-IgG1 (RMG1–1), and FITC-conjugated anti-IgG2a (RMG2a-62). Mouse regulatory T cells (Tregs) were stained using a kit from eBioscience (San Diego, CA, USA, Ca. No. 88–8111). Negative controls comprised conjugated isotype antibodies. A FACScan flow cytometer (Partec Co., Görlitz, Germany) was used to analyze the stained cells. FLOWJo 7.5.5 software was used to analyze the data.

### Quantitative real-time reverse transcription-polymerase chain reaction

On day 4 post-transplantation, the grafts were excised from the recipients. The Trizol reagent (Invitrogen, Carlsbad, CA, USA) was used to isolate RNA from the heart allografts following the manufacturer’s protocol. A ReverTra Ace® qPCR RT Kit (code no. FSQ-101) and a SYBR® Green Realtime PCR Master Mix -Plus- (code no. QPK-212, 212 T) (Toyobo, Japan) were used to perform reverse transcription and PCR, respectively. The StepOne Real-Time PCR System (Applied Biosystems, Carlsbad, CA, USA) was used to perform the data analysis. The normalizing control gene was *Actb* (encoding β-actin), and the reactions were performed three times. Table 2 shows the primers used in the present study.

**Table 2:** qRT-PCR primers used in the present study

Sequences of the primers (5′–3′)		
Target gene	Forward	Reverse
β-actin	CATCCGTAAAGACCTCTATGCCAAC	ATGGAGCCACCGATCCACA
TNF-α	CATCTTCTCAAAATTCGAGTGACAA	TGGGAGTAGACAAGGTACAACCC
IFN-γ	CGGCACAGTCATTGAAAGCCTA	GTTGCTGATGGCCTGATTGTC
IL-2	GGAGCAGCTGTTGATGGACCTAC	AATCCAGAACATGCCGCAGAG
IL-4	TCTCGAATGTACCAGGAGCCATATC	AGCACCTTGGAAGCCCTACAGA
IL-10	GACCAGCTGGACAACATACTGCTAA	GATAAGGCTTGGCAACCCAAGTAA
FOXP3	CAGCTCTGCTGGCGAAAGTG	TCGTCTGAAGGCAGAGTCAGGA
TGF-β	TGACGTCACTGGAGTTGTACGG	GGTTCATGTCATGGATGGTGC
FasL	GCCCATGAATTACCCATGTCC	ACAGATTTGTGTTGTGGTCCTT
Perforin	AACTCCCTAATGAGAGACGCC	CCACACGCCAGTCGTTATTGA
Granzyme B	CCACTCTCGACCCTACATGG	GGCCCCCAAAGTGACATTTATT

### Enzyme-linked immunosorbent assay

On day 4 post-transplantation, serum was sampled from the recipient mice. Commercially available kits (Yikesai Bioproduct Limited Company, Qingpu, Shanghai, China) were used to detect IL-2, IL-10, IL-4, IFN-γ, and TGF-β using ELISA following the manufacturer’s protocol. Each reaction was repeated three times. Known amounts of the purified recombinant murine cytokines were used to construct a standard curve.

### Statistical methods

The Kaplan–Meier method was used to calculate and compare the mean survival times (MSTs) of the four groups. One-way analysis of variance (ANOVA) was used to analyze the data from the flow cytometry, MLR, qRT-PCR, and ELISA experiments, and were expressed as the mean ± SEM. A Bonferroni correction was calculated and applied for multiple comparisons. *P* < 0.05 was taken to indicate statistical significance; *P* < 0.01 and *P* < 0.001 indicate very and extremely significant differences, respectively. GraphPad Prism® software (GraphPad, Inc., La Jolla, CA, USA) was used to perform all the analyses.

## Data Availability

The datasets analysed during the current study are available from the corresponding author on reasonable request.

## Abbreviations

TD: Thalidomide
Abs: Monoclonal antibodies
Tms: Memory T cells
IFN-γ: Interferon gamma
LFA-1: Anti-lymphocyte function-associated antigen 1
ICAM-1: Intercellular adhesion molecule
HTm: Heart transplantation model
MST: Mean survival time
MLR: Mixed lymphocyte reaction
H&E: Hematoxylin and eosin
ELISA: Enzyme-linked immunosorbent assay
qRT-PCR: Quantitative real-time reverse transcription PCR
TNF-α: Tumor necrosis factor alpha
FOXP3: Forkhead box P3
