# Correction: Thalidomide with blockade of costimulatory molecules prolongs the survival of alloantigen-primed mice with cardiac allografts

**DOI:** 10.1186/s12865-022-00512-5

**Published:** 2022-08-09

**Authors:** Maoshu Zhu, Yunhan Ma, Kai Tan, Liyi Zhang, Zhaowei Wang, Yongsheng Li, Yingyu Chen, Junjun Guo, Guoliang Yan, Zhongquan Qi

**Affiliations:** 1 Xiang’an Branch, The First Affiliated Hospital of Xiamen University, Xiamen, 361100 Fujian China; 2 The Fifth Hospital of Xiamen, Xiamen, 361100 Fujian China; 3 Organ Transplantation Institute, School of Medicine, Xiamen University, XiamenFujian, 361100 China; 4 Fujian Key Laboratory of Organ and Tissue Regeneration, Xiamen, 361100 Fujian China; 5 Grade 2015 Clinical Medicine, Fuzhou Medical College of Nanchang University, Fuzhou, 344000 Jiangxi China; 6 School of Medicine, Guangxi University, Nanning, 530004 Guangxi China

## Correction: BMC Immunol (2020) 21:19 10.1186/s12865-020-00352-1

Following publication of the original article [1], the author noticed an error in the funding number available in the Funding section. The funding number of the National Natural Science Foundation of China was incorrectly given as 81771271 and should have been 81771721.

The original article [1] has been corrected.
